# Metabolic and Microvascular Risk Factors Associated With Brain Health in Type 1 Diabetes

**DOI:** 10.1002/acn3.70428

**Published:** 2026-05-12

**Authors:** Jihyun Park, Emily J. Koubek, Richard Beare, Lynn Ang, Brian C. Callaghan, Rodica Pop‐Busui, Eva L. Feldman, Evan L. Reynolds

**Affiliations:** ^1^ Department of Neurology University of Michigan Ann Arbor Michigan USA; ^2^ National Centre for Healthy Ageing, Peninsula Clinical School Monash University Melbourne Australia; ^3^ Department of Internal Medicine, Division of Metabolism, Endocrinology, and Diabetes University of Michigan Ann Arbor Michigan USA; ^4^ Division of Endocrinology, Diabetes and Clinical Nutrition, School of Medicine Oregon Health and Science University Portland Oregon USA; ^5^ Department of Epidemiology and Biostatistics Michigan State University East Lansing Michigan USA

**Keywords:** brain structure, cognition, metabolic factors, microvascular complications, type 1 diabetes

## Abstract

We examined relationships between metabolic factors, microvascular complications, and brain health in adults with type 1 diabetes. Fifty‐one adults were assessed for metabolic risk factors, microvascular complications, and cognitive function, with a subset completing brain MRI. Compared to normative data, participants showed reduced fluid cognition (*p* = 0.01) but elevated crystallized cognition (*p* = 0.0001). Microvascular complications and metabolic risk factors (including obesity, diabetes duration, and HbA1c) were not consistently linked to brain health outcomes. These exploratory findings suggest subtle cognitive changes in adults with type 1 diabetes, though larger longitudinal studies are needed to clarify how metabolic and microvascular factors influence brain health.

## 1 Introduction

Diabetes is a major risk factor for dementia [1]. However, most landmark studies linking mid‐life metabolic risk factors, microvascular complications, and cognitive decline have focused on type 2 diabetes, with fewer examining these associations in type 1 diabetes (T1D) [2, 3, 4]. Research in T1D has also primarily involved younger populations, with limited attention to cognitive decline in midlife and older age, even though patients are now living longer and reaching ages where cognitive impairment becomes clinically significant.

The Diabetes Control and Complications Trial (DCCT) and its follow‐up, the Epidemiology of Diabetes Interventions and Complications (EDIC) study, are the only studies to comprehensively assess how metabolic factors and diabetes‐related complications influence midlife brain outcomes in individuals with T1D. In particular, the DCCT‐EDIC study found severe hypoglycemic episodes, HbA1c, body mass index (BMI), systolic blood pressure (SBP), cholesterol, and chronic kidney disease to be associated with poorer fluid cognition in middle‐aged and older adults with longstanding T1D [5, 6]. Additionally, DCCT‐EDIC found changes in brain structure, with individuals with T1D showing smaller total brain, gray (GMV), and white matter volume (WMV), and larger ventricle and white matter hyperintensity (WMHV) volumes, compared with non‐diabetic controls [7].

The objective of the present study was to assess whether the associations between metabolic factors and microvascular complications with cognitive outcomes and structural brain changes observed in the DCCT/EDIC studies can be replicated in a separate, independent cohort of adults with longstanding T1D, thereby further elucidating key determinants of brain health in this population.

## 2 Methods

### 2.1 Population and Recruitment

Between February 2021–August 2024, 51 adults with T1D were recruited from four previous University of Michigan cohorts of individuals with T1D [8, 9, 10]. All participants completed metabolic and cognitive assessments. Twenty (39%) completed brain MRI sequences. Demographic information included age, sex, race/ethnicity, and education. The University of Michigan Institutional Review Board approved the study (HUM00185622). All participants provided written informed consent.

### 2.2 Metabolic Risk Factors and Anthropometric Measurements

T1D duration (years), height (cm), weight (kg), BMI (kg/m^2^), SBP (mmHg), HbA1c (%), triglycerides (mg/dL), and high‐density lipoprotein cholesterol (HDL; mg/dL) were measured at the cognitive assessment visit.

### 2.3 Microvascular Complications

Diabetic peripheral neuropathy was defined by the Toronto Consensus definition of probable neuropathy [11], retinopathy by the presence of proliferative retinopathy in the worst eye based on review of fundus photos, cardiovascular autonomic neuropathy with the expiration/inspiration (E/I) ratio [8], and chronic kidney disease as having estimated glomerular filtration rate (eGFR) < 60 mL/min/1.73m^2^ [12].

### 2.4 Cognition

Outcome measures of cognition were the NIH Toolbox Cognitive Battery (NIHTB‐CB) fluid cognition composite score and the crystallized cognition composite score. The fluid cognition composite score measures cognitive abilities involved in learning, reasoning, memory, and processing speed, whereas the crystallized cognition composite score reflects accumulated knowledge and verbal skills (e.g., receptive vocabulary and reading recognition) and is often used as an indicator of premorbid intellectual ability or cognitive reserve [13]. The fluid cognition composite score is derived from five individual tests: flanker inhibitory control and attention (measuring attention/executive function), picture sequence memory (episodic memory), list sorting working memory (working memory), dimensional change card sort (executive function), and pattern comparison processing speed (processing speed). Rather than serving as an independent measure, the fluid cognition composite score is calculated based on the relative performance across its subtests, with each contributing proportionally according to a participant's performance. Similarly, the crystallized cognition composite score is derived from two tests, picture vocabulary and oral reading recognition. We also assessed the seven individual tests individually. NIHTB‐CB scores were standardized to normative data (mean = 50, standard deviation [SD] = 10), based on participant age, sex, race/ethnicity, and education level [14].

### 2.5 Brain Structure and Function

MRI scans were acquired using a SIGNA 3 T scanner (GE HealthCare, Chicago, IL). Additional details regarding scan acquisition and processing are provided in **Supplemental Methods**. MRI‐derived brain metrics included cortical thickness, GMV, WMV, subcortical gray matter volume (SGMV), WMHV, median cerebral blood flow (CBF), and fractional amplitude of low‐frequency fluctuations (FALFF).

### 2.6 Statistical Analysis

One‐sample *t*‐tests compared participant cognition to normative data [14]. Normality of the cognition and MRI‐derived metrics was assessed using the Shapiro–Wilk test and Q‐Q plots. Two‐sample *t*‐tests (normal data) or Wilcoxon rank‐sum tests (non‐normal data) compared cognition and MRI‐derived metrics between participants with versus without obesity (defined as BMI≥ 30 kg/m^2^). Pearson's (normal data) or Spearman (non‐normal data) correlations assessed associations between T1D duration, cognition, and MRI‐derived metrics. Linear regression examined associations between metabolic factors or microvascular complications with cognition or MRI‐derived metrics, adjusting for total intracranial volume and age in MRI analyses. WMV, WMHV, and CBF were log‐transformed to account for skewness. Due to the small sample size, analyses were considered exploratory and hypothesis‐generating, with *p* < 0.05 regarded as significant. To control the false discovery rate, the Benjamini‐Hochberg method for multiple comparisons was applied to the linear regression models. Cohen's effect sizes (f^2^) were also calculated to quantify the magnitude of the association (≥ 0.02 small, ≥ 0.15, medium, ≥ 0.35 large) and to aid interpretation beyond statistical significance. Analyses were performed in R (v4.4.1).

## 3 Results

Among the 51 participants with T1D, the mean (SD) age was 56.2 (13.3) years, 45.0% were female, 94% were white, 49% were obese, and 90% had at least a Bachelor's degree (Table 1). Mean T1D duration was 35.1 (14.0) years. Mean BMI was 30.6 (5.5) kg/m^2^ and HbA1c was 7.2 (1.2) % (55.7 [13.2] mmol/mol). Diabetic peripheral neuropathy, retinopathy, and chronic kidney disease were detected in 63%, 32%, and 14% of participants, respectively. The mean E/I ratio was 1.2 (0.4) and the mean eGFR was 82.7 (20.8) mL/min. Participants had significantly higher crystallized cognition (i.e., premorbid intellectual ability or cognitive reserve; Score = 55 [11], *p* = 0.0001) and reading recognition scores (Score = 54 [11], *p* = 0.01) compared to normative data (Table 2).

**TABLE 1:** Demographic information, metabolic factors, and microvascular complications of study participants.

Variable	Mean (SD) or *n* (%)			
	Full cohort (*n* = 51)	Obese (BMI ≥ 30 kg/m^2^) *N* = 25, full	Non‐obese (BMI < 30 kg/m^2^) *n* = 26, full	Cohort with completed MRI (*n* = 20)
Demographic information				
Age, years	56.2 (13.3)	54.7 (13.1)	57.7 (13.6)	54.9 (13.9)
Sex, female	23 (45%)	12 (48%)	11 (42%)	12 (60%)
Ethnicity, Hispanic/Latino	2 (4%)	2 (8%)	0 (0%)	2 (10%)
Race				
White	48 (94%)	23 (92%)	25 (96%)	18 (90%)
Black or African American	1 (2%)	0 (0%)	1 (4%)	0 (0%)
Other	2 (4%)	2 (8%)	0 (0%)	2 (10%)
Education				
High school graduate	5 (10%)	4 (16%)	1 (4%)	
College graduate, bachelor's degree	30 (59%)	14 (56%)	16 (62%)	11 (55%)
Graduate school, professional degree	16 (31%)	7 (28%)	9 (35%)	7 (35%)
History of stroke, yes	2 (4%)	1 (4.0%)	1 (4%)	0 (0%)
Diagnosis of dementia, yes	0 (0%)	0 (0%)	0 (0%)	0 (0%)
Metabolic risk factors				
Type 1 diabetes duration, years	35.1 (14.0)	35.7 (15.0)	34.5 (13.2)	36.3 (14.4)
Height, cm	172.3 (9.8)	172.9 (10.7)	171.7 (8.9)	168.9 (8.0)
Weight, kg	91.5 (20.8)	105.4 (18.0)	78.1 (13.2)	89.1 (18.1)
Body mass index, kg/m^2^	30.6 (5.5)	35.1 (3.7)	26.4 (3.0)	31.1 (5.5)
Adiposity, obese	25 (49%)	—	—	13 (65%)
Systolic blood pressure, mmHg	133.2 (16.8)	133.5 (18.7)	133.0 (15.1)	131.4 (20.1)
Hemoglobin A1c (HbA1c), %	7.2 (1.2)	7.2 (1.0)	7.3 (1.4)	6.9 (0.7)
HbA1c, mmol/mol	55.7 (13.2)	55.2 (11.1)	56.2 (15.2)	52.2 (7.6)
Triglycerides, mg/dL	87.5 (44.5)	107.6 (53.7)	68.1 (19.7)	80.7 (32.9)
High‐density lipoprotein, mg/dL	57.8 (16.5)	52.3 (16.1)	63.1 (15.4)	60.5 (11.0)
Microvascular complications				
Diabetic peripheral neuropathy, yes	32 (63%)	14 (56%)	18 (69%)	11 (55%)
Retinopathy, yes	15 (32%)	8 (36%)	7 (28%)	3 (16%)
Expiration/inspiration ratio	1.2 (0.4)	1.3 (0.5)	1.1 (0.1)	1.2 (0.1)
Estimated glomerular filtration rate, mL/min	82.7 (20.8)	80.5 (23.5)	84.7 (18.1)	86.2 (18.9)
Chronic kidney disease, yes	7 (14%)	5 (20%)	2 (7.7%)	2 (10%)

Abbreviations: BMI, body mass index; MRI, magnetic resonance imaging; SD, standard deviation.

**TABLE 2:** Unadjusted association between obesity, type 1 diabetes duration, cognition and MRI‐derived metrics.

Variable	Overall	*p* ^a^: Type 1 diabetes vs normative data (mean = 50, SD = 10)	Non‐obese (BMI < 30 kg/m^2^) *n* = 26, full *n* = 7, MRI Mean (SD) or Median [range]	Obese (BMI ≥ 30 kg/m^2^) *N* = 25, full *N* = 13, MRI Mean (SD) or Median [range]	*p*: Obese vs non‐obese	Correlation with type 1 diabetes duration	*p*: Correlation with type 1 diabetes duration
**Cognition (*n* = 51)**							
Fluid composite score	47 (9)	**0.01**	45 (5)	48 (11)	0.15^b^	−0.27	0.05^d^
Flanker inhibitory control and attention test (attention/executive function)	52 (15)	0.25	47 [34–75]	46 [23–81]	0.4^c^	−0.17	0.23^e^
Picture sequence memory test (episodic memory)	47 (10)	**0.01**	44 [28–69]	46 [31–75]	0.6^c^	−0.18	0.21^e^
List sorting working memory test (working memory)	52 (9)	0.28	49 (7)	54 (11)	0.09^b^	−0.10	0.47^d^
Dimensional change card sort test (executive function)	48 (12)	0.23	49 (10)	48 (15)	0.72^b^	−0.07	0.63^d^
Pattern comparison processing speed test (processing speed)	47 (13)	**0.01**	42 (10)	51 (14)	**0.01** ^b^	−0.26	0.06^d^
Crystallized composite score	55 (11)	**0.0001**	—	—	—	—	—
Picture vocabulary test (language)	51 (8)	0.45	—	—	—	—	—
Oral reading recognition test (language)	54 (11)	**0.01**	—	—	—	—	—

Abbreviation: BMI, body mass index.

^a^ One‐sample *t*‐test, text in bold indicates *p* < 0.05.

^b^ Two‐sample *t*‐test, text in bold indicates *p* < 0.05.

^c^ Wilcoxon‐rank sum test.

^d^ Pearson's correlation.

^e^ Spearman correlation.

Fluid cognition (i.e., cognitive abilities involved in learning, reasoning, memory, and processing speed) was significantly lower among participants (Score = 47 [9], *p* = 0.01; Table 2) relative to normative data. Among NIHTB‐CB fluid cognition subdomains, participants also demonstrated lower scores in episodic memory (Score = 47 [10], *p* = 0.01) and processing speed (Score = 47 [13], *p* = 0.01). Surprisingly, obesity was associated with better processing speed (non‐obese = 42 [10], obese = 51 [14]; *p* = 0.01). Notably, after adjusting for multiple comparisons using the Benjamini‐Hochberg procedure, no findings remained statistically significant.

Linear regression revealed that higher BMI associated with better fluid cognition (PE = 0.44, 95% CI: 0.02 to 0.86) and faster processing speed (PE = 0.71, 95% CI: 0.08 to 1.35; Table 3). Elevated SBP was the only metabolic factor associated with MRI‐derived metrics, correlating with increased SGMV (PE = 84.99, 95% CI: 8.87 to 161.12). Again, no results were significant after correcting for multiple comparisons.

**TABLE 3:** Adjusted association between metabolic risk factors, cognition, and MRI‐derived metrics.

Variable	BMI (kg/m^2^) PE (95% CI) [*f* ^2^]^a^	HbA1c (%) PE (95% CI) [*f* ^2^]	TG (mg/dL) PE (95% CI) [*f* ^2^]	HDL (mg/dL) PE (95% CI) [*f* ^2^]	SBP (mmHg) PE (95% CI) [*f* ^2^]	Type 1 diabetes duration (years) PE (95% CI) [*f* ^2^]
**Cognition (*n* = 51)**						
Fluid composite score	**0.44** **(0.02, 0.86)** ^b^, ^c^ **[0.09]**	−0.29 (−2.30, 1.73) [0.002]	0.02 (−0.04, 0.07) [0.01]	−0.04 (−0.18, 0.11) [0.01]	−0.13 (−0.27, 0.01) [0.07]	−0.17 (−0.33, 0.002) [0.08]
Flanker inhibitory control and attention test (attention/executive function)	0.46 (−0.30, 1.23) [0.03]	−1.55 (−5.07, 1.98) [0.02]	0.04 (−0.06, 0.14) [0.02]	0.04 (−0.22, 0.30) [0.002]	−0.08 (−0.34, 0.17) [0.01]	−0.13 (−0.44, 0.17) [0.02]
Picture sequence memory test (episodic memory)	0.22 (−0.30, 0.74) [0.02]	0.80 (−1.58, 3.18) [0.01]	−0.02 (−0.09, 0.04) [0.01]	0.11 (−0.07, 0.28) [0.03]	−0.05 (−0.23, 0.12) [0.01]	−0.18 (−0.38, 0.02) [0.07]
List sorting working memory test (working memory)	0.40 (−0.05, 0.85) [0.07]	−1.35 (−3.43, 0.73) [0.04]	0.02 (−0.03, 0.08) [0.02]	−0.04 (−0.19, 0.12) [0.01]	0.004 (−0.15, 0.16) [0.001]	−0.07 (−0.25, 0.12) [0.01]
Dimensional change card sort test (executive function)	0.05 (−0.59, 0.68) [0.001]	0.73 (−2.16, 3.62) [0.01]	−0.05 (−0.13, 0.02) [0.04]	0.09 (−0.12, 0.30) [0.01]	−0.11 (−0.32, 0.10) [0.02]	−0.06 (−0.31, 0.19) [0.01]
Pattern comparison processing speed test (processing speed)	**0.71** **(0.08, 1.35)** **[0.1]**	−1.03 (−4.08, 2.01) [0.01]	0.08 (−0.00, 0.16) [0.07]	−0.13 (−0.35, 0.09) [0.03]	−0.21 (−0.42, 0.00) [0.08]	−0.24 (−0.50, 0.01) [0.07]
**MRI‐derived functional/structural brain metrics (*n* = 20)**						
Cortical thickness, mm	−0.002 (−0.0073, 0.0037)	0.02 (−0.022, 0.068)	−0.0008 (−0.0016, 0.0001)	0.002 (−0.0010, 0.0044)	−0.0005 (−0.0025, 0.0015)	0.00004 (−0.0026, 0.0027)
Gray matter volume, mm^3^	687.95 (−1264.30, 2640.19)	5395.13 (−10894.31, 21684.57)	−123.52 (−458.49, 211.45)	−295.10 (−1289.46, 699.26)	273.03 (−429.93, 976.00)	31.8726 (−898.46, 962.20)
Log (White matter volume, mm^3^)^d^	−0.0004 (−0.004, 0.003)	−0.001 (−0.03, 0.03)	−0.0003 (−0.001,0.0003)	0.001 (−0.001, 0.003)	0.0004 (−0.001, 0.002)	−0.0004 (−0.002, 0.001)
Subcortical gray matter volume, mm^3^	−7.70 (−252.43, 237.02)	−507.22 (−2527.66, 1513.23)	−21.60 (−62.07, 18.88)	60.25 (−59.63, 180.12)	**84.99** **(8.87, 161.12)**	−36.58 (−149.60, 76.44)
Log (White matter hyperintensity volume, mm^3^)	0.02 (−0.02, 0.05)	0.07 (−0.21, 0.35)	0.004 (−0.002, 0.01)	−0.01 (−0.03, 0.007)	0.0001 (−0.01, 0.01)	0.001 (−0.02, 0.02)
Log (Median cerebral blood flow, mL/100 g/min)	0.01 (−0.02, 0.04)	−0.02 (−0.25, 0.21)	−0.002 (−0.01, 0.002)	0.01 (−0.01, 0.02)	0.0001 (−0.01, 0.01)	−0.004 (−0.02, 0.01)
Mean fractional amplitude of low‐frequency fluctuations	−0.0005 (−0.003, 0.002)	0.0049 (−0.012, 0.022)	−0.0002 (−0.0006, 0.0001)	0.0004 (−0.0006, 0.002)	−0.00002 (−0.0008, 0.0007)	0.0008 (−0.0001, 0.0017)

Abbreviations: BMI, body mass index; CI, confidence interval; HbA1c, hemoglobin A1c; HDL, high‐density lipoproteins; PE, point estimate; SBP, systolic blood pressure; TG, triglycerides.

^a^ Cohen's effect sizes (f^2^) were calculated based on the explained variance (R^2^).

^b^ Text in bold indicates statistical significance (*p* < 0.05) based on multivariable linear regression. Each cell represents an individual model. For MRI‐derived metrics, the model was adjusted for total intracranial volume and age.

^c^ No significant results were observed after adjusting *p*‐values using the Benjamini‐Hochberg method.

^d^ White matter volume, white matter hyperintensity, and median cerebral blood flow were log transformed to account for skewness (Shapiro–Wilk test, *p* < 0.05).

No microvascular complications associated with fluid cognition (Table 4). However, diabetic peripheral neuropathy (PE = −12.30, 95% CI: −19.0 to −5.57) and retinopathy (PE = −8.84, 95% CI: −16.80 to −0.88) associated with slower processing speed (Table 4). In contrast, diabetic peripheral neuropathy associated with increased cortical thickness (PE = 0.07, 95% CI: 0.01 to 0.13). Cardiovascular autonomic neuropathy and chronic kidney disease did not associate with MRI‐derived outcomes.

**TABLE 4:** Association between microvascular complications, cognitive metrics, and MRI‐derived metrics.

Variable	DPN (yes/no) PE (95% CI) [*f* ^2^]^a^	RN (yes/no) PE (95% CI) [*f* ^2^]	CAN (E/I ratio) PE (95% CI) [*f* ^2^]	CKD (yes/no) PE (95% CI) [*f* ^2^]
**Cognition (*n* = 51)**				
Fluid composite score	−3.91 (−8.78, 0.96) [0.05]	−5.32 (−10.70, 0.05) [0.09]	−2.70 (−9.24, 3.84) [0.01]	−2.46 (−9.44, 4.53) [0.01]
Flanker inhibitory control and attention test (attention/executive function)	−6.38 (−15, 2.23) [0.05]	−3.62 (−13.3, 6.04) [0.01]	0.79 (−10.9, 12.5) [0.001]	−9.17 (−21.3, 2.92) [0.05]
Picture sequence memory test (episodic memory)	0.54 (−5.38, 6.46) [0.001]	−2.25 (−8.84, 4.33) [0.01]	−3.68 (−11.5, 4.15) [0.02]	−1.46 (−9.78, 6.85) [0.003]
List sorting working memory test (working memory)	−4.39 (−9.48, 0.70) [0.06]	−0.99 (−6.83, 4.85) [0.003]	−1.22 (−8.04, 5.60) [0.003]	0.40 (−6.96, 7.76) [0.001]
Dimensional change card sort test (executive function)	3.52 (−3.59, 10.6) [0.02]	−4.35 (−12.3, 3.57) [0.03]	−5.47 (−14.80, 3.88) [0.03]	−6.99 (−16.9, 2.9) [0.04]
Pattern comparison processing speed test (processing speed)	**−12.30** **(−19, −5.57)** ^b^, ^c^ **[0.28]**	**−8.84** **(−16.80, −0.88)** **[0.11]**	2.08 (−8, 12.2) [0.004]	1.46 (−9.18, 12.1) [0.002]
**MRI‐derived functional/structural brain metrics (*n* = 20)**				
Cortical thickness, mm	**0.07** **(0.01, 0.13)**	−0.06 (−0.17, 0.06)	−0.06 (−0.33, 0.21)	−0.08 (−0.18, 0.02)
Gray matter volume, mm^3^	16,325 (−6751.7, 39,402)	−8401 (−51,433, 34,632)	−14,610 (112,570, 83,347)	−13,463 (−51,758, 24,831)
Log (White matter volume, mm^3^)^d^	0.004 (−0.04, 0.05)	−0.05 (−0.12, 0.02)	−0.08 (−0.25, 0.09)	0.003 (−0.06, 0.07)
Subcortical gray matter volume, mm^3^	911 (−2087.4, 3910)	−3525 (−8437.7, 1387.6)	−4100 (−16,014, 7813.2)	−297 (−5095, 4500)
Log (White matter hyperintensity, mm^3^)	−0.31 (−0.70, 0.07)	0.51 (−0.18, 1.20)	−0.07 (−1.76, 1.61)	−0.04 (−0.71, 0.63)
Log (Median cerebral blood flow, mL/100 g/min)	−0.08 (−0.42, 0.26)	0.07 (−0.50, 0.63)	−1.06 (−2.31, 0.18)	0.35 (−0.15, 0.86)
Mean fractional amplitude of low‐frequency fluctuations	0.01 (−0.01, 0.04)	−0.001 (−0.05, 0.05)	0.02 (−0.08, 0.12)	−0.01 (−0.05, 0.04)

Abbreviations: CI, confidence interval; CAN, cardiovascular autonomic neuropathy; CKD, chronic kidney disease; DPN, diabetic peripheral neuropathy; E/I, expiration/inspiration; PE, point estimate; RN, retinopathy.

^a^ Cohen's effect sizes (f^2^) were calculated based on the explained variance (R^2^).

^b^ Text in bold indicates statistical significance (*p* < 0.05) based on multivariable linear regression. Each cell represents an individual model. For MRI‐derived metrics, the model was adjusted for total intracranial volume and age.

^c^ No significant results were observed after adjusting *p*‐values using the Benjamini‐Hochberg method.

^d^ White matter volume, white matter hyperintensity, and median cerebral blood flow were log transformed to account for skewness (Shapiro–Wilk test, *p* < 0.05).

Cognition correlated with several MRI‐derived metrics. Participants with higher fluid cognition had a thicker cortex (PE = 0.005, 95% CI: 0.0002 to 0.01), greater GMV (PE = 1789.07, 95% CI: 477.43 to 3100.72), and greater SGMV (PE = 240.056, 95% CI: 28.31 to 451.81; Table S1). Higher scores on dimensional change card sort test associated with greater GMV (PE = 841.65, 95% CI: 114.42 to 1568.88).

## 4 Discussion

In this contemporary cohort of adults with longstanding T1D, we investigated the relationships between metabolic factors, microvascular complications, and brain health outcomes. In concert with prior studies that reported that T1D increases the risk of cognitive decline [5, 6] we found that the T1D participants evaluated experienced reductions in fluid cognition, episodic memory, and processing speed. However, although some of these differences reached statistical significance, cognitive performance generally remained within the normal range (i.e., less than one standard deviation [10] below the population mean [50]), suggesting that these reductions may not necessarily reflect clinically meaningful impairment.

Surprisingly, diabetes duration and HbA1c were not consistently linked to brain health outcomes, which may reflect limited statistical power. Another possibility is that our cohort was actively involved in clinical research studies and thus had relatively well‐controlled disease (mean HbA1c = 7.2 [1.2] %), reducing detectable effects. Importantly, with 90% of our cohort holding at least a college degree, although the NIHTB‐CB scores adjust for education level, it's possible that higher education attainment allowed participants to become resilient to the negative impacts of longstanding diabetes on cognitive performance during this age‐period. Indeed, higher education has been shown to protect against cognitive decline in type 2 diabetes (T2D) [15, 16, 17]. Future work in populations with lower education levels is needed to determine whether the effect of longstanding diabetes has differential importance across education levels.

Midlife metabolic factors and microvascular complications have also been suggested as drivers of brain health in diabetes. Surprisingly, in this study, metabolic factors were not consistently associated with brain health outcomes, and when associations were observed, some ran counter to expectations. For example, participants with obesity demonstrated faster processing speeds, higher BMI associated with better fluid cognition, and elevated SBP correlated with increased SGMV. These results contrast with previous studies which generally reported that obesity and hypertension were associated with poorer cognition [5]. However, after correcting for multiple comparisons, these associations were no longer statistically significant, raising the possibility that these findings may reflect false positive results given our small sample size and the large number of statistical comparisons. Consistent with this interpretation, effect sizes from the regression analyses were generally small (Cohen's f [2] < 0.15), indicating modest effect magnitudes and suggesting limited clinical impact. We also did not account for comprehensive midlife risk factors, such as physical activity, depression, hearing loss, or smoking status. Interestingly, similar paradoxical findings have been observed in T2D, where obesity correlated with better cognition [18]. These results highlight the complex relationship between BMI and brain health, suggesting that further longitudinal studies are needed to clarify how BMI impacts brain health across the lifespan.

No microvascular complications associated with fluid cognition, though prior studies have reported such links [19], although diabetic peripheral neuropathy and retinopathy were associated with slower processing speed. Unexpectedly, diabetic peripheral neuropathy was also positively associated with cortical thickness, though prior studies reported decreased thickness with diabetic peripheral neuropathy [20]. Given that associations between microvascular complications and brain health are largely due to the presence of shared risk factors, it is possible that these microvascular complications occur first, and changes to cognition have yet to occur. Given these unexpected results, additional longitudinal studies are needed to elucidate the role midlife microvascular complications have on future brain health.

Finally, several cognitive domains are associated with MRI‐derived metrics. Specifically, higher cognitive scores correlated with a thicker cortex, greater GMV, and sGMV. Also, Dimensional change card sort test associated with greater GMV. These findings align with previous research demonstrating associations between cognition and neuroimaging markers in both T1D and T2D populations [7], suggesting that costly and time‐intensive MRIs may not always be necessary.

Limitations include a small, self‐selected sample size, particularly for MRI metrics, a large number of statistical comparisons, a highly educated cohort (limiting generalizability to less‐educated populations), lack of comprehensive dementia risk factor assessment, and absence of a matched control group without diabetes. Additionally, after correcting for multiple comparisons, none of the results remained statistically significant, suggesting that findings should be interpreted as exploratory and hypothesis‐generating.

In summary, although adults with longstanding T1D demonstrated subtle cognitive reductions and associations between cognition and brain structure, the lack of significant findings after correction for multiple comparisons highlights the need for larger, longitudinal studies in more diverse populations to clarify how midlife metabolic and microvascular factors influence brain health in T1D.

## Author Contributions

B.C.C., R.P.B, E.L.F., and E.L.R. were involved in the conception of the study. E.L.R. and J.P. designed and analyzed the study. All authors interpreted the results. J.P. and E.J.K. wrote the first draft of the manuscript. All authors edited, reviewed, and approved the final version of the manuscript. E.L.R. is the guarantor of this work and, as such, had full access to all the data in the study and takes responsibility for the integrity of the data and the accuracy of the data analysis.

## Funding

This work was supported by the Richard and Jane Manoogian Foundation. NeuroNetwork for Emerging Therapies at the University of Michigan. Andrea and Lawrence A. Wolfe Brain Health Initiative. Robert E. Nederlander Sr. Program for Alzheimer's Research. Robert and Katherine Jacobs Environmental Health Initiative. Dr. John H. Doran Neuropathy Research Initiative. Juvenile Diabetes Research Foundation Center of Excellence at University of Michigan (5‐COE‐2019–861‐S‐B). National Institutes of Health (R00DK129785, R01DK129320, R01DK130913).

## Disclosure

Prior Presentation: Data within this article was presented as a poster at the 2025 American Diabetes Association Scientific Sessions.

## Conflicts of Interest

The authors declare no conflicts of interest.

## Supporting information


**Table S1:** Association between cognitive and MRI‐derived brain metrics.

## Data Availability

The data that support the findings of this study are available from the corresponding author upon reasonable request.
