# Supplementary material for: Metabolic and Microvascular Risk Factors Associated With Brain Health in Type 1 Diabetes
Source: Ann Clin Transl Neurol. 2026 May 12;13(9):1937–46. doi: 10.1002/acn3.70428 (PMC13394883; doi:10.1002/acn3.70428)
Supplement: Supplementary file 1 [file ACN3-13-1937-s001.docx]

**SUPPLEMENTAL METHODS**

**Brain structure and function**

T1 weighted structural images (1mm isotropic, 208x256x256 voxels, SPGR, TE=2.9ms, flip angle = 8degrees, TI = 1060ms, TR=2500ms) were processed with FreeSurfer 7.4 [1] to segment the cortical surface and measure global brain morphometrics including mean cortical thickness (mm), total cortical and subcortical grey matter and total white matter volume (mm^3). White matter hyperintensities were segmented using BIANCA applied to FLAIR (sagittal acquisition, 1x1x2mm, 256x256x90 voxels, TE=140ms, flip angle=90 degrees, TI=1073ms, TR=6000ms) scans [2]. Resting state MR data (2.4mm isotropic 90x90x60 voxels, TE=30.0ms, flip angle = 52deg,  TR=800ms) was pre-processed using fmriprep [3], with motion correction performed using independent component analysis with automatic removal of motion artefacts (ICA-AROMA) and the DPABI toolbox was used to compute fractional amplitude of low frequency fluctuation (fALFF) scores with a low frequency range of 0.01-0.08Hz [4-6]. fALFF scores are a unitless ratio between signal power in the low frequency range to the total signal power. Perfusion weighted scores were derived from arterial spin label scans scans (voxels 1.875x1.875x4mm, 128x128x30 voxels, TE=52ms, flip angle = 111 degrees, TR=4760ms) using the vendor software (Release: RX28.0_R04). The sequence did not include calibration scans and the resulting image was unitless. fALFF and perfusion scores were projected to the cortical surface created by FreeSurfer and global means computed.

**REFERENCES**

[1]	Fischl B, Dale AM (2000) Measuring the thickness of the human cerebral cortex from magnetic resonance images. Proc Natl Acad Sci U S A 97(20): 11050-11055. 10.1073/pnas.200033797

[2]	Griffanti L, Zamboni G, Khan A, et al. (2016) BIANCA (Brain Intensity AbNormality Classification Algorithm): A new tool for automated segmentation of white matter hyperintensities. Neuroimage 141: 191-205. 10.1016/j.neuroimage.2016.07.018

[3]	Esteban O, Markiewicz CJ, Blair RW, et al. (2019) fMRIPrep: a robust preprocessing pipeline for functional MRI. Nat Methods 16(1): 111-116. 10.1038/s41592-018-0235-4

[4]	Pruim RHR, Mennes M, van Rooij D, Llera A, Buitelaar JK, Beckmann CF (2015) ICA-AROMA: A robust ICA-based strategy for removing motion artifacts from fMRI data. Neuroimage 112: 267-277. 10.1016/j.neuroimage.2015.02.064

[5]	Zou QH, Zhu CZ, Yang Y, et al. (2008) An improved approach to detection of amplitude of low-frequency fluctuation (ALFF) for resting-state fMRI: fractional ALFF. J Neurosci Methods 172(1): 137-141. 10.1016/j.jneumeth.2008.04.012

[6]	Yan CG, Wang XD, Zuo XN, Zang YF (2016) DPABI: Data Processing & Analysis for (Resting-State) Brain Imaging. Neuroinformatics 14(3): 339-351. 10.1007/s12021-016-9299-4

**SUPPLEMENTAL TABLES**

**Table S1. Association between cognitive and MRI-derived brain metrics**

|  | **Fluid composite score**  **PE (95% CI)** | **Flanker inhibitory control and attention test (attention / executive function)**  **PE (95% CI)** | **Picture sequence memory test (Episodic memory)**  **PE (95% CI)** | **List sorting working memory test (working memory)**  **PE (95% CI)** | **Dimensional change card sort test (executive function)**  **PE (95% CI)** | **Pattern comparison processing speed test (processing speed)**  **PE (95% CI)** |
| --- | --- | --- | --- | --- | --- | --- |
| **Mean fractional amplitude of low-frequency fluctuations** | 0.001  (-0.001, 0.002) | 0.0004  (-0.0004, 0.001) | -0.0004  (-0.0015, 0.0007) | -0.0001  (-0.002, 0.001) | 0.0001  (-0.0007, 0.0008) | 0.001  (0.0000, 0.001) |
| **Cortical thickness** | **0.005***  **(0.0002, 0.01)** | -0.0005  (-0.003, 0.002) | 0.004  (-0.0001, 0.0073) | -0.004  (-0.009, 0.0002) | 0.002  (-0.0002, 0.005) | 0.001  (-0.002, 0.004) |
| **Log(Median cerebral flow)** | 0.015  (-0.001, 0.031) | 0.001  (-0.009, 0.011) | 0.012  (-0.0004, 0.03) | -0.001  (-0.019, 0.016) | 0.004  (-0.005, 0.013) | 0.002  (-0.008, 0.012) |
| **Gray matter Volume** | **1789.07***  **(477.43, 3100.72)** | 178.409  (-694.3, 1051.12) | 979.33  (-200.8765, 2159.5358) | -1240.123  (-2681.96, 201.72) | **841.65***  **(114.42, 1568.88)** | 455.16  (-412.64, 1322.96) |
| **Log(White matter volume)** | 0.002  (-0.0001, 0.0041) | 0.0003  (-0.0010, 0.0016) | 0.001  (-0.0005, 0.0030) | -0.001  (-0.003, 0.001) | 0.0002  (-0.0011, 0.0014) | 0.001  (-0.001, 0.002) |
| **Subcortical gray matter volume** | **240.056***  **(28.31, 451.81)** | 58.42  (-72.51, 189.34) | 156.398  (-22.77, 335.57) | -48.563  (-288.37, 191.24) | 69.89  (-54.42, 194.20) | 78.748  (-52.73, 210.22) |
| **Log(White matter hyperintensity volume)** | -0.014  (-0.04, 0.01) | -0.0001  (-0.014, 0.014) | -0.014  (-0.033, 0.005) | 0.02  (-0.002, 0.042) | -0.002  (-0.02, 0.011) | -0.005  (-0.02, 0.01) |

* Each cell indicates a linear regression model adjusted with ICV, text in bold indicates *p*<0.05

* Pearson’s correlation coefficient, text in bold indicates *p*<0.05
